# Implications of built and social environments on the academic success among African American youth: testing Strong African American Families intervention effects on parental academic racial socialization

**DOI:** 10.3389/fpsyg.2023.956804

**Published:** 2023-08-16

**Authors:** Velma McBride Murry, Catherine M. Gonzalez, Marlena L. Debreaux, Erica E. Coates, Cady Berkel

**Affiliations:** ^1^Department of Human and Organizational Development, Department of Health Policy, School of Medicine, Peabody College, Vanderbilt University, Nashville, TN, United States; ^2^Department of Human and Organizational Development, Vanderbilt University, Nashville, TN, United States; ^3^Department of Psychiatry, MedStar Georgetown University Hospital/Georgetown University School of Medicine, Washington, DC, United States; ^4^College of Health Solutions, Arizona State University, Tempe, AZ, United States

**Keywords:** family-based prevention, academic racial socialization, academic success, school climate, academic disparities

## Abstract

Studies exploring widening academic disparities have highlighted the role of racialized school settings, which have given way to incidents of discrimination and unfair treatment for students of color, disproportionately affecting African American youth. Research also shows that family-based preventive interventions may avert negative outcomes for this population through the promotion of protective socialization practices. Consequently, the current study tests the efficacy of a culturally tailored preventive family-based program to foster induced changes in academic promotive parenting practices that prepare youth to advance academically by navigating negative race-related experiences in school settings. Data collected over four time periods from the Strong African American Families (SAAF) efficacy trial (Murry and Brody, 2004) with 667 African American families in rural Georgia were used for this study. Structural equation modeling analyses demonstrated that the SAAF program was associated with positive intervention induced changes in parental academic race-related socialization, which in turn, was indirectly associated with reduced school compromising behaviors through the enhancement of racial pride. While discrimination compromised academic success, our findings highlight the protective nature of racial pride in dissuading academic failure and school dropout through the promotion of academic success. This study confirms that a family-based prevention program holds promise to address academic disparities through the enhancement of parenting and youth protective processes that buffer youth from succumbing to racialized social environments such as schools. Implications for research, educational policy, and preventive interventions are discussed.

## Introduction

The foundational role of schools in the community has been established through multiple systems that are structurally and functionally integrated and embedded in history and sociocultural systems, including educational, public policy, governmental and economic systems. Studies examining the effects of school climate and social environment have focused on ways in which social interactions and the quality of one’s surroundings or physical environment impacts human development and behavior (Stewart et al., 2021). Thus, studies of schools as social environments have explored ways in which teacher and peer relationship quality, school policies, as well as parental school involvement predict youths’ academic aspirations and school performance (Marchand et al., 2014; Kwong and Davis, 2015; Berkowitz et al., 2017). Negative school experiences have been identified as drivers of academic disparities, with notable detriment for African American youth, who are among the most vulnerable of populations experiencing educational marginalization in the United States (Morris and Perry, 2017; Causadias and Umaña-Taylor, 2018; Gaylord-Harden et al., 2018; Welsh and Little, 2018; Jones et al., 2021). It has also been established that family-based preventive interventions may avert negative outcomes for this population through the promotion of protective practices such as academic and racial socialization (Murry et al., 2019; Berkel et al., 2022). The goal of the current study was to determine whether a strengths-based and culturally centered, family-based preventive intervention would be effective in enhancing parenting processes that encourage their children to thrive academically, by buffering them from succumbing to the negative consequences of race-related social interactions experienced in school. In the following section we provide a brief overview of studies that informed the conceptualization of our study.

### Schools as racialized social environments

While school should be a place of safety for African American youth, it is often a social setting where stressful race related experiences begin as early as pre-kindergarten, followed by continued episodes of being scapegoated by educational systems as uneducable because of disruptive, aggressive, and oppositional behaviors displayed in school settings (Gibbons et al., 2004; Murry et al., 2009; Dumas, 2016). Race related experiences can occur through numerous social interactions, including peers, teachers, authority figures, or the internet. These race related experiences can be manifested as microaggressions, implicit biases, or explicit and vicarious verbal and physical incidents of discrimination fueled by systems and structures of inequalities (English et al., 2020). One of the consequences of growing up in a racialized society is the negative characterization and social marginalization of African American students, which set forth perceptions of low expectations about their aptitude, scholastic capacities, intellectual competence, and overall morality (Chin et al., 2020). Race related experiences and negative stereotyping of African American students in school settings begin as early as pre-K.

Several studies report significant levels of racial discrimination as early as age 3–4 years old (Caughy et al., 2004) which increases in frequency as African American children age (Fisher et al., 2000; Gibbons et al., 2004; Sellers et al., 2006). For example, recent research indicates that, on average, African American adolescents experience over five racial discrimination experiences a day (English et al., 2020), with girls and boys undergoing similar racialized experiences in schools. While much attention has been given to the ill effects of racial discrimination on the plight of African American males’ school experiences, several studies have also shown that African American girls are also at risk for disproportionate detentions, suspensions, and expulsions (Young and Butler, 2018). African American boys, for example, were twice as likely to be recipients of harsh disciplinary practices and infractions than White boys; however, African American girls were three times more likely to experience inequitable disciplinary practices compared to White girls (Morris and Perry, 2017). Out-of-school suspension and expulsion practices, in particular, were often driven by teachers’ negative stereotypes of African American youth, characterizing boys as “unteachable,” aggressive, and violent (James, 2012). Girls, on the other hand, were often described as sassy, loud, and defiant toward teachers and school personnel (Cooper et al., 2022). These stereotyped perceptions of teachers are thought to be major contributors to not only disparities in school disciplinary practices, but ultimately drivers of widening academic disparities for African American youth, through reduced academic engagement (Larson et al., 2019).

### Academic failure and school dropout

A plethora of studies have documented association between race related experiences and school outcomes. For example, several studies found that pervasive acts of implicit racial attitudes, as well as implicit bias and explicit acts of racial discrimination from teachers toward African American students have been associated with African American students’ academic failure and school dropout (Wang and Huguley, 2012; Chin et al., 2020). Further, being subjected to punitive disciplinary practices, including detentions, suspensions, expulsions, and assignments to alternative schools or special education classes (Skiba et al., 2002) have been associated with academic failure. Confronted with a sense of “otherness” from teachers and school professionals, African American students may cope with racial discrimination by disengaging cognitively and physically from schools to avoid negative race-related encounters (Murry et al., 2009). Moreover, some students may engage in self-protection behaviors, namely *academic self-presentation*, often camouflaging their academic ability, and potentially jeopardizing their academic performance and school success (Ogbu,1992; Murry et al., 2009; Cooper et al., 2022). Other students, who experience teacher racialized victimization, may physically disengage through increased school absences, heightened social isolation and alienation, academic deterioration, and eventually, dropping out of school (Strange et al., 2012; Leath et al., 2019). To cope with these negative social interactions, many African American students engage in coping behaviors that may become a self-fulfilling prophecy, such when students disengage academically, it confirms teachers’ negative stereotyped perceptions of their academic potential. Given that many African American youth do not succumb to race related adversities, it is important to identify factors and processes that explain how they are able to successfully navigate these seemingly insurmountable odds to become successful contributing members of society.

### Parents and prosocial peers as academic influencers

Several studies have demonstrated the protective nature of African American parents’ capacity to socialize their children in a positive way, through which youth learn to be aware of racism and reject negative societal messages about their race (Murry et al., 2009; Wang and Huguley, 2012). In fact, powerful factors that protect African American youth originate in the family, particularly in caregiving practices (Brody et al., 2005). For example, African American parents, recognizing the likelihood that their children will be exposed to disparate treatment in schools due to racism and discrimination, socialize and motivate their children to do well in school (Hill et al., 2004; Murry et al., 2009). Socialization practices to prepare youth for racial bias and marginalization have not only been shown to increase awareness of discrimination and “othering,” but also to promote youths’ capacity to reject negative societal messages about one’s race, and thereby succeed (Chavous et al., 2003; Murry et al., 2009). Cultural socialization, or teaching youth to identify with and take pride in their cultural backgrounds has been consistently associated with improved academic outcomes (Hughes et al., 2006; Neblett et al., 2006; Wang et al., 2020). For example, previous research found that cultural socialization attenuated the effects of teacher discrimination on grade point averages and educational aspirations (Wang and Huguley, 2012). According to Wang and Huguley (2012), preparing youth of color for racial discrimination and teaching them to embrace their racial heritage buffered the negative influence of race-related experiences in schools and in turn, dissuaded school failure. The protective nature of adaptive racial socialization for rural African American youth’s school success has also been observed (Murry et al., 2009).

In fact, many African American families have important strengths that foster resilience and, in turn, help their children develop into competent individuals (Murry et al., 2018). Positive parent–child relationships that are emotionally and instrumentally supportive are protective and foster the enhancement of emotional regulation and cognitive control capacities among youth (Morris et al., 2017). These internal self-management processes have been associated with behaviors that foster academic success, including planful decision-making, capacity and skills for paying attention, avoiding distraction, setting priorities, controlling impulses, and recognizing, understanding, and accepting emotions (Hughes and Dexter, 2013). Moreover, attentive, involved, and vigilant parenting also teaches children to anticipate and avoid potentially disadvantageous situations, such as affiliation with deviant peers (Simons et al., 2002; Murry et al., 2011).

Studies of academic outcomes fail to extensively capture the positive contributions of peers. Gonzales et al. (1996) reported that peers provide opportunities for students to formulate study habits, academic self-concept, academic motivation, and attitudes and perceptions regarding school importance and academic and career achievement. Further, academically oriented peers have the potential to counter the harmful effects of stereotype threat in educational settings, in turn, reducing maladaptive coping used to protect one’s integrity and self-academic performance (Murry et al., 2009). In addition, prosocial peer affiliation has also been identified as a protective factor, weakening the association between racial discrimination and not only risk for underperforming academically, but also dissuading engagement in disruptive behaviors (Brody et al., 2006; Murry et al., 2009).

Collectively, we coined the term, *academic racial socialization*, to capture ways in which African American parents engage in practices to increase racial awareness, sense of racial group belongingness, pride in their history and ancestry, and the importance of setting goals to succeed in school. We contend that academic racial socialization practices may be employed by parents to socialize their children in ways to recognize and prepare for race related experiences in a manner that reduces the likelihood of consequences (e.g., harsh disciplinary strategies) that may compromise their academic success. Academic racial socialization is based on the theoretical orientation and empirical studies linking parents’ ethnic-racial socialization to positive youth outcomes (Hughes et al., 2006; Murry et al., 2009). This construct specifically focuses on parenting that African Americans engage in to not only foster positive identity development in their children but to also promote academic success and lifelong success. It is worth highlighting that despite the widening academic disparity gap, limited attention has been given to identifying ways in which African American parents and families prepare their children to navigate experiences in schools that lead to academic success (Murry et al., 2009). To address this void, there is a need for prevention scientists to harness protective processes in African American families that help their children navigate interactions in schools that may be drivers of negative academic outcomes.

In sum, while school climate and social environment may perpetuate structural inequalities that contribute to academic disparities among African American youth, we hypothesize that parents and peers may serve a protective and promotive role to deter youth from succumbing to race-related school challenges. Further, results of a randomized trial demonstrated that a cluster of intervention-targeted parenting strategies, which included racial socialization plus universal parenting skills related to parent–child relationships and positive discipline, were associated with improvements in adolescent racial pride and multiple behavioral health outcomes (Brody et al., 2005; Murry et al., 2005, 2007, 2009, 2011, Berkel et al., 2022). The current study was conducted to expand on previous preventive intervention findings by testing the extent to which a family-strength based program, the Strong African American Families (SAAF) program, designed to deter risk engaging behaviors among rural African American would demonstrate efficacy in promoting academic success, through a cluster of parenting strategies, characterized as academic racial socialization.

SAAF causative model, a theory of the individual and family processes that explain the program’s effects on rural African American youths’ engagement in sexual risk behavior (Murry et al., 2005), is based on several theories. Bronfenbrenner and Morris (1998) and Cicchetti and Toth (1997) conceptions of the ecology of development,  Zimbardo and Boyd (1999) time perspective theory, Bandura’s (1997) and Barkley’s (1997) theories about the development of self-regulatory mechanisms, and Gibbons and Gerrard’s (1997) prototype/willingness model of adolescent health risk behavior. Specific processes were selected for inclusion in the model on the basis of Murry and Brody’s prior and ongoing longitudinal research with rural African American youth and their families (Murry and Brody, 1999, 2002; Brody et al., 2004, 2005). Our causative model was based on an expectation that exposure to the SAAF curriculum would evince positive youth developmental outcomes through program-related effects on caregivers’ parenting processes which we conjectured would foster youths’ intrapersonal protective processes. We hypothesize that parental academic racial socialization will foster protective processes that encourage youth to take pride in their racial identity, invest in their education, and subsequently avoid masking their academic potential as a way of coping with the negative perceptions of teachers and non-accepting peers (Murry et al., 2009). We further hypothesize that academic competence, which we conjecture represents prosocial peer affiliation, motivation to do well in school, positive attitudes about school, as well as positive relationship with teacher, will play a key role in positively orienting youth academically, thereby protecting them from academic failure and school dropout. Recognizing that race related experiences can occur early in the life of African American youth with implications for later development, we also sought to examine how exposure to protective familial factors in middle childhood would have prognostic significance for academically promotive behaviors, in spite of discriminative experiences, that in turn increase academic success as youth transitioned into adolescence. We selected these developmental stages as they have been identified as critical periods when youth are met with increased peer influence (García Coll and Szalacha, 2004) and a time when African American youth are more likely to receive expulsion or out of school experiences (Skiba et al., 2011).

## Conceptual framework

**Figure 1:**
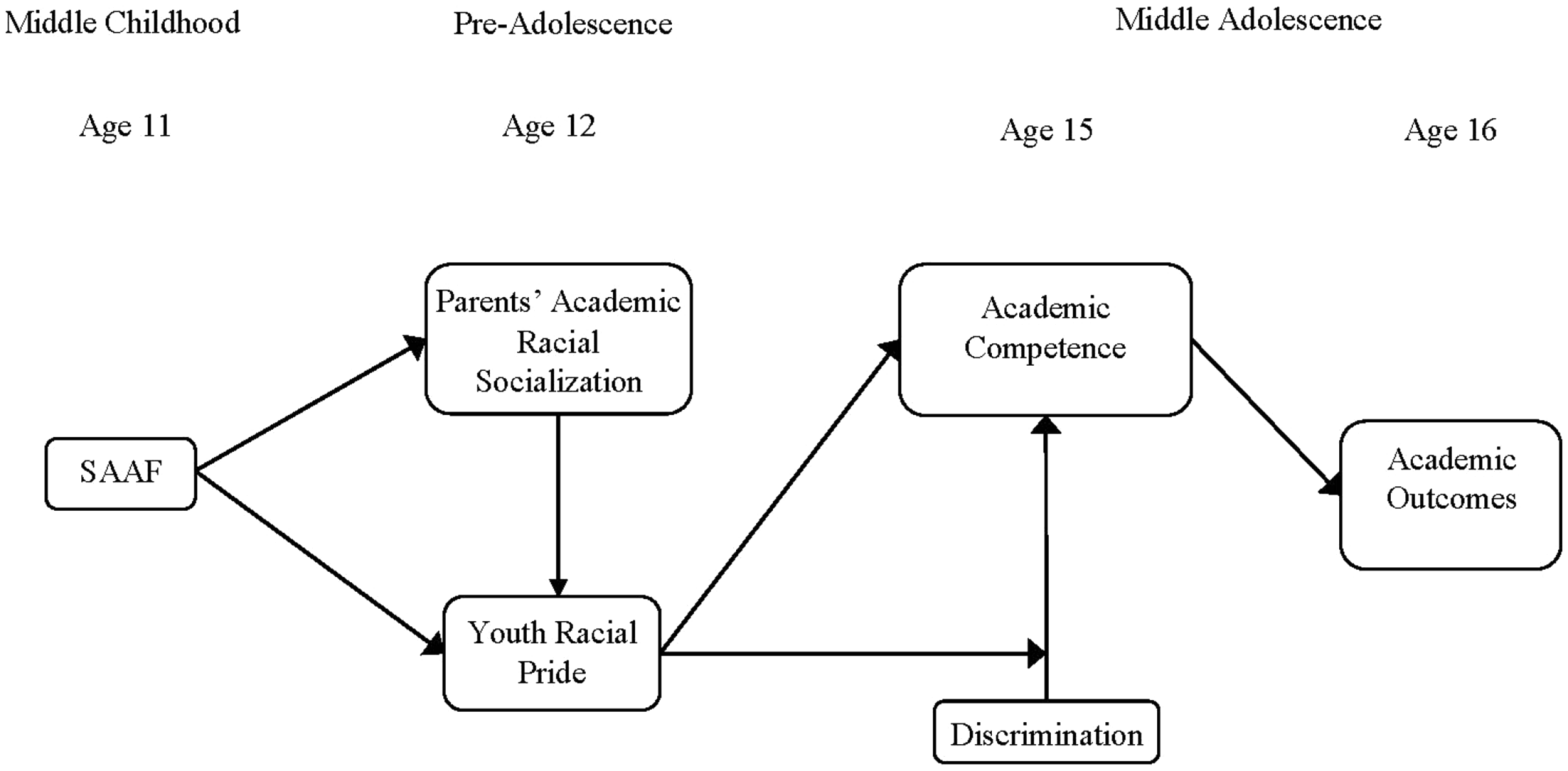
Conceptual Model (Murry et al., 2018).

To inform and guide our hypotheses, both Bronfenbrenner (1977) and Murry et al.’s (2018) integrative model for the study of stress within African American families were selected as our conceptual frameworks. The ecological model is based on an assumption that human development is impacted by dynamic relational interactions that are inextricably linked with and infused into multiple interlocking contextual systems. Humans are not only influenced by the social environments around them but are active agents employing capacities and skills to influence, as well as be influenced by environments (e.g., Tudge et al., 2009). To illustrate this process, Murry et al.’s (2018) model posits that the stress endured by African American families is, in part, a by-product of systemic racism (See Figure 1). Illuminated in this model are strengths-based, cultural assets that African Americans use to navigate socio-environmental contextual stressors, by enhancing close relationships in families, which foster positive developmental outcomes in youth. Further, this model characterizes these strengths-based, cultural assets as “ordinary magic” (Masten, 2001) which may explain how African American parents and families foster positive academic performance and aspirations in their children by assuaging the negative consequences of racialized upstream practices that have been shown to derail and hinder optimal development and adjustment potential for their children. Murry’s model also acknowledges how social relationships, such as prosocial peer affiliation, can encourage behaviors that promote successful life transition, including school completion. In the following section, we provide a summary of the SAAF program, to test its efficacy in fostering academic racial socialization and in turn promoting academic success.

## Overview of the Strong African American Families program

The Strong African American Families (SAAF) parenting program was developed specifically for use with rural African Americans. It is based on longitudinal, developmental research with this population and feedback from community stakeholders (Brody et al., 2004), through which proximal and malleable processes were identified in youths’ immediate family contexts that facilitate academic and social competence and inhibit risk behaviors. This research indicates that powerful factors protecting children and adolescents from behavior problems originate in the family, particularly in parents’ caregiving practices (Berkel et al., 2009; Murry et al., 2009). Supportive relationships with parents foster self-regulation, academic and social competence, psychological adjustment, and avoidance of alcohol use and early sexual onset among rural African American youth (Brody et al., 2006a; Murry et al., 2011). A cluster of specific protective parenting processes, which we termed *regulated-communicative parenting*, were identified in our research program, and targeted in SAAF. This cluster includes consistent discipline, parental monitoring, and open communication. SAAF also targeted youth intrapersonal protective factors, which include academic competence, social competence, and self-regulation. We expected increases in these factors to result directly from intervention attendance and to be sustained over time by changes in parenting behavior.

Strong African American Families’s efficacy was evaluated in a randomized prevention trial with 667 rural African American families. The intervention’s efficacy and the validity of the causative model on which it was based were supported. Families who participated in SAAF experienced increases in regulated-communicative parenting (Brody et al., 2004, 2005; Murry et al., 2005) and in youth intrapersonal protective processes (Brody et al., 2004; Murry et al., 2005). SAAF-induced effects on parenting behavior not only enhanced deterrents to adolescent risk behavior, such as negative attitudes toward risk behaviors (Murry et al., 2005), future orientation (Brody et al., 2004), self-regulation (Brody et al., 2005), and resistance efficacy (Brody et al., 2004), but also inhibited the early onset of substance use and sexual intercourse (Brody et al., 2006a) and dampened alcohol use trajectories (Brody et al., 2006b). It is not known, however, whether the program is effective in enhancing parenting strategies to reduce academic disparities. In the present study, we sought to capitalize on the implementation effectiveness of SAAF beyond the targeted outcomes and determine the non-targeted benefits of the program on other high-risk behavioral outcomes confronting African American families, such as low academic performance of African American students.

## Methods

### Data, participants, and procedures

In the original sample of the SAAF Program, a total of 667 families were randomly selected to participate in the study, resulting in a recruitment rate of 65%. The retention rate for families providing data in all data across time periods was greater than 84%. In 53.6% of these families, the target child was female. All families resided in Georgia, and the youth, both males and females, averaged 11.2 years of age at the time of enrollment. The families who completed the study had an average of 2.7 children. In terms of household composition, 33.1% of parents were single, 23.0% were two-parent, married households, 33.9% were married but separated from their partner, and 7.0% were living with partners to whom they were not married. Of the two-parent families, 93.0% included both target child’s biological parents, with mothers representing the majority of participating parents. Caregivers/mothers’ mean age was 38.1 years. Most of the mothers, 78.7%, had completed high school. The family median household income was $1,655.00 per month.

To identify pathways through which exposure to a family-based preventive intervention during middle childhood may forecast academic performance during the pre-adolescence and middle-adolescence stages, we selected data points that include baseline (Time 1; Age 11), 6 months’ post-intervention (Time 2; Age 12), 48 months’ post-intervention (Time 4; Age 15), and 54 months’ post-intervention (Time 5; Age 16). Of the 667 families at baseline, 571 families remained in the study at Time 6, and attrition rates were similar by treatment condition (IV: 88%, Control: 82%).

## Measures

### Group assignment to SAAF

Random assignment in SAAF was dummy coded, with participation in the intervention group coded as 1 and participation in the control group coded as 0.

### Parental academic racial socialization

An eight-item scale measure was developed using seven items from the Racial Socialization Scale (Hughes and Johnson, 2001) and one item from the General Child Management Survey (Spoth et al., 1998) to assess parents/caregivers’ approaches and strategies to prepare their children to navigate race-related experiences in school settings. Examples of items include, “*I know the way that I handle racism teaches my children how to handle these situations*” and “*How often in the past month have you told your child that he/she must be better than White kids to get the same rewards because of his/her race?*,” using a three-point Likert scale ranging from 1 *(never)* to 3 *(three to five times)*, *𝛼* = 0.79 across both Times 1 and 2.

### Youth racial pride

During middle childhood and pre-adolescence, youth responded to an 8-item questionnaire, the Inventory of Black Identity (Sellers et al., 1997), using a 5-point Likert scale ranging from 1 *(strongly disagree)* to 5 *(strongly agree)*. Statements included “*Being Black is an important part of my self-image,”* and “*I am happy to be Black,*” with 𝛼 = 0.60 and 0.67, for Times 1 and 2, respectively.

### Youth academic success

Youths’ academic success consists of indicators form an adapted version of Harter’s (1982) academic engagement and competence scale and affiliating with prosocial academically oriented peers (adapted from Elliott et al., 1985). For the academic orientation measure, 20 items were rated on a five-point Likert scale ranging from 1 *(Strongly disagree)* to *(Strongly agree).* The measure was composed of four subscales—motivation to do well in school, self-efficacy completing assignments, positive attitudes toward school, and relationship with teachers—and included sample items such as, “Grades are very important to me” and “I feel very close to at least one of my teachers.” Responses to the items were re-coded and summed to obtain a total score for each indicator to reflect youth academic success, with 𝛼 = 0.80 for all indicators across middle adolescence (Time 1: 𝛼 = 0.85; Time 2: 𝛼 = 83). Affiliating with prosocial academically oriented peers was measured using a 13 items scale, in which youth responded to a three-point Likert scale ranging from 1 *(all of them)* to 3 *(none of them) to items such as:* “During the past 12 months…” with a sample statement including “how many of your close friends have skipped school without an excuse?” Cronbach’s 𝛼 was 0.80 across data collections.

### Racial discrimination

Youth rated their exposure to race-related experiences, using the 9-item Racist Hassles Questionnaire (Harrell, 1997), were rated on a 4-pt scale from 1 *(never)* to 4 *(frequently)*. Example items were, “*blamed for something or treated suspiciously (as if you have done something or will do something wrong) because of your race,*” “*treated rudely or disrespectfully because of your race*,” and “*called a name or harassed because of your race*.” Cronbach’s *α* was 0.90.

### Academic risk outcomes

Two indicators from the Personal Life Stressor (Holmes and Rahe), measure were used to assess academic failure and dropout vulnerability. For academic failure, youth were asked if in the past 12 months, they had failed in school on a 3-point Likert scale: 1 *(no)*, 2 *(yes)*, or 3 *(not in school)*. To measure school dropout, youth were asked, “*In the past 12 months, did you quit or drop out of school?*,” using a two-point Likert scale (1 = *no*, 2 = *yes*).

### Analytic plan

Study hypotheses were tested via structural equation modeling in Mplus 8.1 (Muthén and Muthén, 2018) in two separate models (see details below). To address missing data, we conducted Little’s (1988) MCAR test, which demonstrated that data were likely missing completely at random (*Χ*^2^(30) = 26.16, *p* = 0.67). Full Information Maximum Likelihood was used to address missing data (Enders and Bandalos, 2001). Nesting of individuals within clusters can result in violations of independence. Because only one of the three conditions was conducted in a group format, we determined that county was the most appropriate cluster variable. ICCs for each variable were all under 0.05, indicating independence of the data by county (Kreft and de Leeuw, 1998).

Multiple fit indices were used to evaluate the adequacy of model fit: either a non-significant *χ*^2^ or a combination of SRMR below 0.08, RMSEA below 0.08, and/or CFI above 0.90, based on simulation studies that revealed using this combination rule resulted in low Type I and Type II error rates (Hu and Bentler, 1999). We also examined modification indices to determine whether additional paths were indicated by the data. The significance of standardized βs represent tests of study hypotheses. We used bias corrected bootstrap confidence intervals to assess the significance of the standardized indirect effects. Specifically, we tested for (a) an indirect program effect on adolescent racial pride through the enhancement of parental academic racial socialization and (b) an indirect protective role of youth racial pride in reducing risk for academic failure and school dropout through the enhancement of academic success. Mediation was considered significant if the 95% CI did not cross zero (MacKinnon et al., 2002; Fritz and MacKinnon, 2007; Taylor et al., 2008).

## Results

### Preliminary analyses

Parental academic racial socialization was associated with youths’ racial pride at ages 11 and 12 (See Table 1). Racial pride during those developmental stages was positively associated with academic competence at age 15. All five indicators of academic competence were significantly intercorrelated (all *p*-values ≤ 0.001). Moreover, academic failure was positively associated with school dropout. None of the other variables were associated with school dropout. Exposure to racial discrimination at 15 years old was negatively associated with academic competence and positively associated with academic failure at 16 years old but not related to school dropout.

**Table 1:** Correlations for study variables.

Variable	1	2	3	4	5	6	7	8	9	10	11	12
1. Parental academic racial socialization-Time 1	–											
2. Racial pride-Time 1	0.08^*^	–										
3. Parental academic racial socialization-Time 2	0.43^**^	0.04	–									
4. Racial pride-Time 2	0.09^*^	0.32^**^	0.12^**^	–								
*Academic competence*:												
5. Affiliation with academically oriented prosocial peers	−0.08	0	−0.01	−0.01	–							
6. Motivation	−0.04	0.09^*^	−0.02	0.11^*^	0.30^**^	–						
7. Self-efficacy	−0.05	0.08	−0.03	0.12^**^	0.27^**^	0.71^**^	–					
8. Positive attitudes	0.02	0.07	0.04	0.03	0.33^**^	0.59^**^	0.57^**^	–				
9. Relationship with teacher	−0.05	0.05	−0.05	0.01	0.34^**^	0.61^**^	0.50^**^	0.53^**^	–			
10. Discrimination	0.10^*^	0.03	0.07	−0.03	−0.31^**^	−0.16^**^	−0.13^**^	−0.21^**^	−0.25^**^	–		
11. Academic failure	0.00	−0.04	0	−0.07	−0.08	−0.12^**^	−0.17^**^	−0.13^**^	−0.14^**^	0.11^*^	–	
12. School drop out	−0.02	−0.02	0.05	−0.01	−0.08	−0.07	−0.08	−0.09	−0.03	0.05	0.25^**^	–
Mean	1.89	4.05	1.93	4.17	2.62	4.30	4.07	4.04	4.16	1.40	1.36	1.05
Standard deviation	0.44	0.94	0.43	0.82	0.36	0.56	0.58	0.60	0.77	0.39	0.64	0.21

^*^p < 0.05; ^**^p < 0.01; and ^***^p < 0.001.

### Test of the hypothesized model

**Figure 2:**
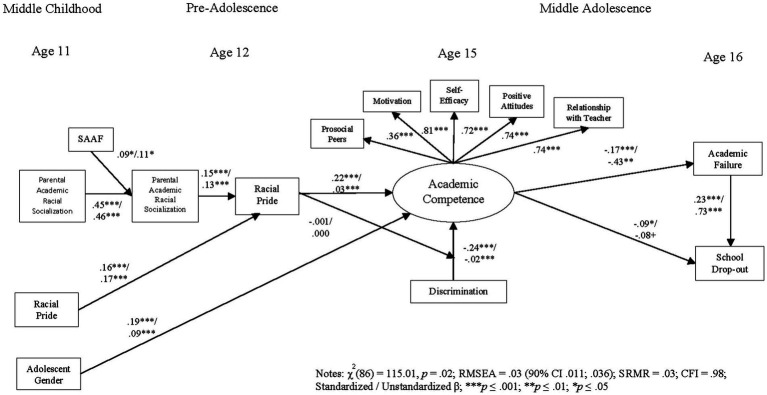
Final analytic model.

Results demonstrated good overall model fit [*χ*^2^ (86) = 115.01, *p* ≤ 0.01; RMSEA = 0.025 (90% confidence interval, CI = 0.011, 0.036), CFI = 0.98]. Standardized *β*s represented tests of study hypotheses (see Figure 2). Loadings for the five indicators of the academic competence latent construct were all above 0.35, *p* ≤ 0.001. We ran different models, testing alternative paths; however, none of the additional paths fit the data as well as the original hypothesized model.

Assignment to SAAF, while their children were in middle childhood, led to improvements in parents’ academic racial socialization (*β* = 0.09, *p* < 0.05), which was consequently positively associated with youths’ racial pride (*β* = 0.15, *p* < 0.001) during pre-adolescence. In turn, youths’ racial pride at age 12 predicted increases in academic competence in middle adolescence (age 15; *β* = 0.22, *p* < 0.001), including motivation to do well in school, association with prosocial peers, heightened sense of efficacy to complete assignments, positive attitudes about school, and positive relationships and interactions with teachers. Exposure to racial discrimination at age 15 predicted decreases in academic competence (*β* = −0.24, *p* ≤ 0.001), and at age 16, academic failure predicted school dropout (*β* = 0.23, *p* ≤ 0.001). Mediational analyses demonstrated a significant indirect program effect on adolescent racial pride through the enhancement of parental academic racial socialization [*β* = 0.15 (95% CI = 0.006, 0.223)]; youth racial pride demonstrated a significant indirect negative association linking academic failure and school dropout mediated through academic competence [*β* = −0.17 (95% CI = 0.006, 0.223) and *β* = −0.09 (95% CI = 0.006, 0.223), respectively]. Finally, we conducted gender moderation analyses, and although there were no significant gender differences in the relationships between parental academic racial socialization and youths’ racial pride, females demonstrated higher academic competence than their male counterparts (*β* = 0.19, *p* < 0.001) during middle adolescence. In sum, random assignment to the SAAF program directly increased parental academic racial socialization strategies, which indirectly influenced academic outcomes of their children through racial pride and academic competence enhancement, which favored daughters more than sons.

## Discussion and conclusions

A plethora of studies have been undertaken to explain the widening academic gap between African American students and their White counterparts. Emerging from those studies is the conclusion that African American students are among the most vulnerable populations experiencing educational marginalization in our society (Venzant Chambers and McCready, 2011; Causadias and Umaña-Taylor, 2018; Gaylord-Harden et al., 2018). While several plausible explanations have been offered, one school of thought is that the racialized experiences of African American students in schools situate their everyday experiences in a context in which social interactions and relationships with teachers, peers, educational policies, and practices impede aspirations, sense of belonging, performance and ultimately, school completion.

Academic disparities are often attributed to teachers’ implicit racial biases and negative subjective evaluations of African American students, often associated with stereotypical images of African Americans. These negative perceptions are often met with discriminatory practices and social interactions and are thought to be contributors to disparate academic outcomes (Chin et al., 2020). While the sources of racism are structural and systemic, requiring radical changes to eliminate social determinants that perpetuate inequities (e.g., education, economics, health and health care, neighborhood and built environment, social and political contexts), African American parents are confronted with challenges to identify ways to navigate the downstream effects of racism on their children’s daily lives, and engage in practices to protect their children from succumbing to adversities (Murry et al., 2009).

In the current study, we test the efficacy of the Strong African American Families program, a strength and culturally centered, family-based preventive intervention program, for its potential promise for enhancing parenting processes, namely academic racial socialization, to facilitate positive school outcomes for children. To our knowledge, this is the first empirical test of a strengths-based, culturally tailored preventive intervention designed to address racial academic achievement gap. Further, we expanded the previous findings of SAAF to concurrently consider the contributions of both parents and prosocial peers as untapped positive influencers of youth developmental outcomes, in this instance academics.

Several key findings emerged from our study. Results suggest that increases in parental academic racial socialization, for those randomly assigned to SAAF, was indirectly associated with preventing academic failure and subsequently, school dropout through its effect on facilitating youth racial pride. This finding expands studies demonstrating the powerful protective nature of parental racial and ethnic socialization practices in dissuading risk-engaging behaviors through the promotion of ethnic-racial identity (Neblett et al., 2006; Huguley et al., 2019). The relevance of the findings from the current study also adds to Byrd and Chavous’ (2009) study, which demonstrated that high racial pride was a strong predictor of high GPA for African American youth, and especially for those living in low resourced communities. Despite raising their children in economically and socially racialized conditions, many rural African American families have important strengths that help their children thrive academically and become competent individuals, despite exposure to negative environments, including schools.

The urgent need for expanding this line of research, protective nature of parental academic racial socialization, is further supported as our findings revealed ways in which racial discrimination can derail African American youths’ academic success. Specifically, our findings demonstrated that racial discrimination was negatively associated with all indicators of youths’ academic outcomes, displaying reduced motivation to do well in school, compromised sense of academic self-efficacy, more negative attitudes toward school, and lowered sense of bonding and connection with teachers. These findings are consistent with previous research demonstrating the detrimental consequences of African American students’ experiences of racial discrimination on academic outcomes (Gale, 2020; Gale and Dorsey, 2020).

Studies of academic disparities have consistently found gender differences, such that girls report greater school achievement, higher school completion, and overall higher educational attainment than males (Duckworth and Seligman, 2006; Chavous and Cogburn, 2007). However, reasons for these differences have not been fully clarified. We sought to add insight on this phenomenon. Further, we also examined whether changes in males and females’ academic outcomes differed across developmental stages. In terms of gender effects, moderation tests were conducted on each of our predicted paths and only one significant finding emerged. At age 11, a critical developmental and academic transitional period, African American girls scored higher on academic competence than boys. While other studies have shown similar findings, our study was designed to test theories that identify specific pathways that explain why girls outperform boys on academic indicators and what promotes change in those outcomes. Both parental academic racial socialization and racial pride were key protective mechanisms that explained these gender differences, favoring higher academic outcomes for girls. That gender differences did not emerge for the remaining paths suggests that the effects of parental academic racial socialization as a protective process against the consequences of racial discrimination for African American youths’ academic outcomes are similar for males and female students. Given this, there is utility for gaining greater insight on the link between racial discrimination and academic competence through narrative descriptions of these experiences. While both male and female African American students are subjected to stereotyped perceptions and characterization by teachers (Larson et al., 2019), we offer a plausible explanation for males’ academic outcomes. Specifically, studies have shown gender inequities that increase educational vulnerability for African American male students across all stages of their development (Thomas and Stevenson, 2009; Howard, 2013; Curry, 2017; Seaton and Tyson, 2019). As early as preschool, African American males are exposed to pervasive and persistent acts of discrimination, deficit framing, and are characterized as a threat to society. These perceptions also influence social interactions with teachers and peers and affect all aspects of their development, including academic outcomes. Smith-Bynum et al.’s (2014) study examining longitudinal patterns of racial discrimination of African American youth, in grades 7–10, found increased episodes of discrimination for African American males, especially in 10th grade. Continued study of these gender effects as well as identification of protective mechanisms is warranted. Our study expands Smith-Bynum et al.’s (2014) investigation by identifying and testing mechanisms that predict academic outcomes across critical developmental stages. To our knowledge, this is among the first study to examine ways in which an evidence, family, and strengths-based program can enhance processes in families to prepare African American youth for experiences that may compromise academic outcomes, with a focus on potential programmatic effects across critical developmental stages, from pre-adolescence to mid-adolescence. Findings from our study may hold promise for reducing the widening academic disparities gap, with implications for research, policies, and the design of integrative family and school-based programs specifically targeting antiracism and the enhancement of school climates and environments that are just and humane for all students.

## Limitations

Although this study has many strengths, there are also limitations to note when interpreting the findings. First, while a strength of the study is the construction and testing of a newly developed measure, parental academic racial socialization, it was developed from two existing measures and as such, warrants further testing to establish internal and external validity. In addition, our measure of racial discrimination assessed overall race-related experiences, not unique to school settings. Recent studies of racial discrimination, for example, utilize more specific measures that directly measure teacher-student relationship quality, through observations that capture social interactions in context (e.g., discrimination from teachers). This approach offers opportunities to examine the nuances of discriminatory experiences in school contexts and their implications for students’ development across multiple domains, including academics (Alliman-Brissett and Turner, 2010; Benner and Graham, 2013; Gale and Dorsey, 2020). Nonetheless, our measure of parental academic racial socialization was highly correlated with all targeted outcomes and racial discrimination was highly correlated with all five factors of academic competence. Academic competence predicted both academic failure, and school dropout. In addition, we acknowledge the extent to which our measure of racial pride, 𝛼 = 0.60 and 0.67, for Times 1 and 2, respectively, under-or overestimate its true reliability. Prior studies have noted that African American adolescents in more racially homogenous environments, as the families included in the current study, may have greater difficulty conceptualizing ideologies of being a minority (Scottham et al., 2008). Our findings, however, do demonstrate an increase in the reliability on this measure after exposure to the SAAF program, suggesting that the implementation of culturally tailored program that promote parents’ academic racial socialization can foster racial pride and in turn contribute to youths’ academic success.

Finally, some of the study pathways were concurrent, rather than longitudinal. The decision about which time points to include was driven by the higher priority and theoretical explanations underlying the SAAF program in terms of its effect in intervention-targeted parenting and youth interpersonal protective processes. That is, we conjectured that parental academic racial socialization and racial pride during preadolescence would lay a foundation to deter the negative effects of discrimination on youth during middle adolescence when problem behaviors and disengagement from school emerge.

## Implications

Despite these limitations, findings from our study have several implications. First, there is a need for more longitudinal efficacy or effectiveness studies that include strengths-based, cultural assets to specify how and for whom protective mechanisms are effective for addressing academic disparities. Studies are needed to disentangle the timing and sequencing of events at various ecological levels. Further, there is a need for more mixed methods research to gain greater insight on factors and processes that explain gender differences in academic outcomes. Future studies that consider academic racial socialization as a context-specific process in families are needed as well as study designs that specifically capture the unique contribution of peers as academic influencers, beyond our proxy of measuring youths’ reports of affiliating with prosocial peers.

In addition, there is a need for multi-level preventive interventions designed to address academic disparities that specifically target parents, youth, peers, teachers, and schools, as each of these systems play a central role in reducing the widening academic disparities gap. A core component of preventive interventions is addressing ways to eradicate structures, policies and practices that create, perpetuate, and maintain structural and systemic racism (Murry et al., 2018).

Our findings also have great implications for school-level policies that differentially impact the educational experiences of African American youth. School-level policies, such as zero-tolerance disciplinary strategies, are often based on implicit racial biases and subjective evaluation of students’ behavior by racially biased school personnel and have disproportionately affected African American youth, contributing to high suspension and expulsion rates. This study calls for policies that ensure that schools are a place of safety that are racially and ethnically affirming social environments. Doing so will require training of school personnel in cultural humility to eradicate stereotypical thinking about African American students. Learning new ways of interaction and teaching, replacing characterizations of “unteachable,” aggressive, and violent (James, 2012; Kunesh and Noltemeyer, 2019), and sassy and loud students (Cooper et al., 2022), with more affirming, accepting and promotive characterizations. These negative subjective perceptions are thought to be major contributors to disparities in school disciplinary practices and compromised school functioning, including early school leaving/dropout, regardless of gender (Leath et al., 2019). Our findings echo the urgent need to address the harm that can occur from local and state-level policies that are banning discussions of race and racism in schools and removing racial and ethnic affirming content and information from educational materials. Therefore, addressing academic disparities requires changing systems that are beyond the reach of families through the removal of upstream barriers that produce and sustain inequities.

## Data availability statement

The raw data supporting the conclusions of this article will be made available by the authors, without undue reservation.

## Ethics statement

The studies involving humans were approved by Vanderbilt University Institutional Review Board. The studies were conducted in accordance with the local legislation and institutional requirements. Written informed consent for participation in this study was provided by the participants’ legal guardians/next of kin.

## Author contributions

VM, CG, and MD contributed to the conceptualization and design of the manuscript. CG and VM organized the database. CG ran preliminary analyses. CB performed the statistical analysis. VM and EC wrote the first draft of the manuscript. CG and VM wrote the methods and results section of the manuscript. VM and MD wrote discussion and implications of the manuscript. All authors contributed to the article and approved the submitted version.

## Funding

This study was supported by the National Institute of Mental Health (MH063043; VM).

## Conflict of interest

The authors declare that the research was conducted in the absence of any commercial or financial relationships that could be construed as a potential conflict of interest.

## Publisher’s note

All claims expressed in this article are solely those of the authors and do not necessarily represent those of their affiliated organizations, or those of the publisher, the editors and the reviewers. Any product that may be evaluated in this article, or claim that may be made by its manufacturer, is not guaranteed or endorsed by the publisher.
